# Investigation of drugs affecting hypertension in bevacizumab‐treated patients and examination of the impact on the therapeutic effect

**DOI:** 10.1002/cam4.3587

**Published:** 2020-11-24

**Authors:** Kenta Yagi, Marin Mitstui, Yoshito Zamami, Takahiro Niimura, Yuki Izawa‐Ishizawa, Mitsuhiro Goda, Masayuki Chuma, Kimiko Fukunaga, Takahiro Shibata, Shunsuke Ishida, Takumi Sakurada, Naoto Okada, Hirofumi Hamano, Yuya Horinouchi, Yasumasa Ikeda, Hiroaki Yanagawa, Keisuke Ishizawa

**Affiliations:** ^1^ Clinical Research Center for Developmental Therapeutics Tokushima University Hospital Tokushima Japan; ^2^ Department of Clinical Pharmacology and Therapeutics University of Tokushima Graduate School of Biomedical Sciences Tokushima Japan; ^3^ Department of Pharmacy Tokushima University Hospital Tokushima Japan; ^4^ Department of Pharmacology University of Tokushima Graduate School of Biomedical Sciences Tokushima Japan

**Keywords:** bevacizumab, colorectal cancer, FAERS, gene expression omnibus, hypertension, proton pump inhibitor

## Abstract

**Background:**

In patients treated with bevacizumab, hypertension may be a biomarker of therapeutic efficacy. However, it is not clear whether drugs that control blood pressure influence bevacizumab's efficacy. In this study, we investigated drugs that may affect hypertension in bevacizumab‐treated patients and examined the impact on the therapeutic effect.

**Patients and methods:**

We analyzed 3,724,555 reports from the third quarter of 2010 to the second quarter of 2015. All data were obtained from the Food and Drug Administration (FDA) Adverse Event Reporting System (FAERS) analysis. In this retrospective cohort study, we investigated a total of 58 patients diagnosed with colorectal cancer and treated for the first time with bevacizumab containing XELOX or mFOLFOX6 at The University of Tokushima Hospital between January 2010 and December 2015. The effect of the treatment was evaluated according to Response Evaluation Criteria in Solid Tumors version 1.0. Thereafter, the effect was confirmed using Gene Expression Omnibus (GEO) and cultured cells.

**Results:**

There are few reports in FAERS of hypertension in patients treated with omeprazole on bevacizumab. Based on the chart review, patients who used proton pump inhibitors (PPI) had a lower response to treatment than those who did not (response rate: 25% vs 50%). Furthermore, experiments on GEO and cell lines suggested that induction of vascular endothelial growth factor (VEGF) gene expression by PPIs is the cause of the reduced therapeutic effect.

**Conclusion:**

PPIs prevent hypertension in bevacizumab‐treated patients but may reduce bevacizumab's anti‐tumoral effects by inducing VEGF expression.

## Graphical abstract

The use of PPIs prevents hypertension in bevacizumab‐treated patients. However, it may reduce the anti‐tumor effect of bevacizumab by inducing the expression of VEGF.

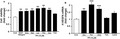

## 1 INTRODUCTION

Bevacizumab is a humanized monoclonal antibody against the vascular endothelial growth factor (VEGF) that inhibits tumor growth by inhibiting angiogenesis. It has shown antitumor effects in several cancer types, including colorectal cancer, breast cancer, non‐small cell lung cancer, renal cell carcinoma, ovarian cancer, glioblastoma, and metastatic melanoma.^1^, ^2^, ^3^ Bevacizumab has been used in combination with chemotherapy, and has been reported to prolong both overall survival and progression‐free survival.^4^, ^5^, ^6^ However, bevacizumab causes a variety of adverse events, one of which is hypertension. One clinical study reported that after bevacizumab treatment, 49.3% of patients had induced‐grade 2, or higher hypertension.^3^

Hypertension induced by bevacizumab administration is thought to occur due to decreased endothelial cell regenerative capacity and decreased production of vasodilators, such as nitric oxide and prostacyclin.^7^ VEGF inhibition leads to the reduction of nitric oxide synthesis, thus reducing the levels of nitric oxide in the body,^7^ which in turn leads to constriction of the blood vessels, and a reduction in sodium ion renal excretion, ultimately resulting in hypertension.^8^ The development of bevacizumab‐induced hypertension is one of the most important problems in continuing the bevacizumab treatment, and even deaths have been reported due to the development of hypertension. If bevacizumab‐induced hypertension develops, bevacizumab should be stopped immediately until blood pressure control is achieved.^9^, ^10^, ^11^

However, several retrospective studies have suggested that among patients treated with bevacizumab, those who developed hypertension had a better prognosis than those who did not.^8^, ^12^, ^13^, ^14^, ^15^ Indeed, in patients with metastatic colorectal cancer who received combination chemotherapy with bevacizumab, overall response, progression‐free survival, and overall survival were associated with the emergence of hypertension.^13^ These studies suggest that the appearance of hypertension may be a biomarker of treatment efficacy in patients treated with bevacizumab. In addition, it is possible that drugs reducing the occurrence of hypertension in patients receiving bevacizumab might have a negative effect on the efficacy of bevacizumab treatment, however, their associations are currently not clear. Most patients treated with bevacizumab use several concomitant drugs. Among these concomitant drugs, there might be several drugs that could alter the blood pressure, but to the best of our knowledge, there are no studies on the interactions of these drug combinations. This could be probably explained partly by the fact that concomitant medications are so varied that it would be difficult to adequately study them in studies involving a small number of centers.

The Food and Drug Administration (FDA) Adverse Event Reporting System (FAERS) is the world's largest database created by the U.S. FDA to monitor the safety risks of approved drugs. Adverse events related to drug use are voluntarily reported to FAERS by patients, physicians, pharmacists, and other health care providers. Currently, millions of drug‐related adverse events are registered in FAERS, which in recent years has supported several academic studies on drug safety and development that are separate from the FDA’s objective of monitoring drug adverse events. For example, FAERS analysis has shown that concomitant drug B reduces the risk of adverse events caused by drug A.^16^, ^17^ Additionally, the Gene Expression Omnibus (GEO) is a global data repository of next‐generation sequencing, microarray, and high‐throughput functional genomics data submitted by research communities.^18^ It is also an integrated database for systematic analyses of gene function and linking gene information with higher‐order functional information.^19^ We have previously analyzed large information databases, such as FAERS and GEO to identify novel drug effects of existing drugs.^20^, ^21^, ^22^

These databases have enabled us to analyze the effects of concomitant drugs, and to estimate the mechanisms of their effects, which otherwise cannot be analyzed in a few centers due to the inadequate number of cases. Therefore, in this study, we aimed to investigate drugs that could alter the incidence of hypertension in bevacizumab‐treated patients and examine whether these drugs could affect the treatment efficacy.

## 2 RESULTS

### 2.1 FAERS analysis for determining the relationship between bevacizumab and hypertension

The FAERS database was used to investigate the occurrence of hypertension in patients treated with bevacizumab. Of 36,95,466 patients, hypertension was reported in 85,079 patients and in 1,520 using bevacizumab. The reporting odds ratio (ROR) for bevacizumab use and hypertension was 2.45, 95% confidence interval (CI): 2.33–2.59, *p* < 0.001, which indicated a strong relationship. A similar relationship was found when the patients were stratified by sex and age (Table 1). Bevacizumab was found to be associated with an increased incidence of hypertension in FAERS, regardless of age or sex.

**Table 1:** Association between bevacizumab and the occurrence of hypertension in FDA Adverse Event Reporting System.

Group	Hypertension without bevacizumab (%)	Hypertension with bevacizumab (%)	ROR	95%CI	*p*‐value
Total	83559/3695466 (2.26)	1520/28288 (5.37)	2.45	2.33‐2.59	<0.001
Stratified by gender					
Male	28076/1274753 (2.2)	414/11466 (3.61)	1.66	1.51‐1.84	<0.001
Female	51603/2090097 (2.47)	776/13658 (5.68)	2.38	2.21‐2.56	< 0.001
Stratified by age (y)					
<40	8342/527869 (1.58)	60/1232 (4.87)	3.19	2.46‐4.14	<0.001
40~4	7758/321558 (2.41)	104/1998 (5.21)	2.22	1.82‐2.71	<0.001
50~59	13054/470547 (2.77)	214/4062 (5.27)	1.95	1.7‐2.24	<0.001
60~69	14250/472096 (3.02)	299/5476 (5.46)	1.86	1.65‐2.09	<0.001
70~100	15478/529240 (2.92)	248/4663 (5.32)	1.86	1.64‐2.12	<0.001

### 2.2 FAERS‐based search for drugs reducing hypertension in patients treated with bevacizumab

The FAERS was used to identify concomitant drugs that were associated with a reduced incidence of hypertension in patients treated with bevacizumab. Epirubicin, Erlotinib, and omeprazole were found to be associated with a lower rate of hypertension in patients treated with bevacizumab (Table 2) in the FAERS analysis. However, among them, patients using omeprazole reported the highest incidence of hypertension.

**Table 2:** Concomitait drugs that decrease occurrence of bevacizumab‐induced hypertension in FDA Adverse Event Reporting System

Drug B	Hypertension without drug B (%)	Hypertension with drug B (%)	ROR	95% CI	*p*‐value
EPIRUBICIN	1512/27859 (5.43)	8/429 (1.86)	0.33	0.16‐0.67	<0.001
ERLOTINIB	1486/27390 (5.43)	34/898 (3.79)	0.69	0.49‐0.97	0.035
OMEPRAZOLE	1502/27692 (5.42)	18/596 (3.02)	0.54	0.34‐0.87	0.008

### 2.3 Demographic and clinical characteristics of patients by examining their medical records

Between January 2010 and December 2015, 62 patients from The Tokushima University Hospital were treated with bevacizumab in combination with XEROX or mFOLFOX6, among whom 58 were included in the analysis. Table 3 shows the patients’ characteristics in the PPI (n = 16) and non‐PPI (n = 42) groups. There were no significant differences between the two groups regarding median age at diagnosis, sex, performance status (PS), histopathological staging, concomitant regimens, or concomitant antihypertensive medications (Table 3).

**Table 3:** Baseline patient characteristics and Responses Categories in the RECIST.

	Non‐ PPI group (n = 42)	PPI group (n = 16)	*p*‐ value
**Median age, y (range)**	68 (40‐86)	62 (42‐82)	0.188^a^
**Gender, n (%)**			
Male	21 (50)	10 (63)	0.5765^b^
Female	21 (50)	6 (38)	
**Stage**			
Ⅰ	1 (2)	0	0.8241^c^
Ⅱ	1 (2)	0	
Ⅲa	5 (12)	1 (6)	
Ⅳ	35 (83)	15 (94)	
**ECOG Performance Status, n (%)**			
0	21 (50)	8 (50)	0.7633^b^
1	23 (48)	7 (44)	
2	0 (2)	1 (6)	
**Combined regimes**			
XELOX	21 (42)	12 (75)	0.1551^b^
mFOLFOX6	21 (42)	4 (25)	
**Anti hypertensive**			
Use	7 (17)	3 (19)	1^c^
Non‐use	35 (83)	13 (81)	
**Response**			
CR	0	0	
PR	21	4	
SD	17	7	
PD	4	5	
Objective control rate	50%	25%	0.138^c^
Disease control rate	90%	69%	0.097^c^

Abbreviations: CR, complete response; PD, progression dessease; PR, partial response; SD, stable disease.

^a^ Mann‐Whitney *U* test.

^b^ Chi‐square test.

^c^ Fisher's exact test.

### 2.4 Influence of PPI‐use on the effectiveness of chemotherapy, including bevacizumab

Response rates were measured. No patient had complete response (CR), 25 had partial response (PR), 24 had stable disease (SD), and 9 had progressive disease (PD). The response rate was 50% in the non‐PPI group and 25% in the PPI group; objective response rate (ORR) was 25% higher in the non‐PPI group than that in the PPI group, with no significant difference. The disease control rate was 50% in the non‐PPI group and 25% in the PPI group. The disease control rate of the non‐PPI group was 21% higher than the PPI group but not significant (Table 3).

In both groups, the blood pressure increased after bevacizumab treatment. The change in mean systolic blood pressure before and after bevacizumab treatment was 13.55 mmHg in the non‐PPI group and 13.94 mmHg in the PPI group, with no significant difference (Table 4).

**Table 4:** Systolic blood pressure before and after bevacizumab treatment in non‐proton pump inhibitors (PPI) and PPI groups.

	mean systolic blood pressure (SD)		
	before treatment with bevasizumab [mmHg]	after treatment with bevasizumab [mmHg]	*p*‐ value
Non‐PPI group	130 (15.33)	143 (19.55)	0.979^a^
PPI group	120 (5.39)	134 (4.56)	

^a^ Mann‐Whitney *U* test.

### 2.5 Evaluating changes in signaling pathways and gene expression associated with the pathway of hypertension using the GEO and Kyoto Encyclopedia of Genes and Genomes (KEGG) databases

To investigate the mechanism of effect of bevacizumab in patients using omeprazole, we analyzed a GEO‐deposited toxicogenomics microarray dataset (GEO Association No. GSE59927). Using these data sets, we analyzed the differences in gene expression in rats treated orally with omeprazole (30 mg/kg, for 5 days) (Table 5). GEO analysis showed that *VEGF* gene expression was upregulated in the rat livers after oral omeprazole treatment.

**Table 5:** Gene Expression Omnibus data showing the effects of omeprazole on the expression levels of vascular endothelial growth factor signaling‐related genes.

Gene title	Omeprazole	Corn Oil	*p*‐value
vascular endothelial growth factor	11.57	11.07	0.048
SPHK1 (sphingosine kinase type 1) interacting protein (Hs.) (DBSS)	5.05	5.69	0.003
neuroblastoma RAS viral (v‐ras) oncogene homolog	7.31	7.53	0.006
v‐raf‐1 murine leukemia viral oncogene homolog 1	10.52	11.01	0.012
nuclear factor of activated T‐cells, cytoplasmic, calcineurin‐dependent 4	4.44	6.54	0.000
ras‐related C3 botulinum toxin substrate 1	10.79	10.43	0.005
nitric oxide synthase (DBSS)	5.23	5.78	0.001

n = 32 for vehicle; n = 3 for omeprazole‐treated group.

### 2.6 Effect of PPI on VEGFA expression using human cell lines

**Figure 1:**
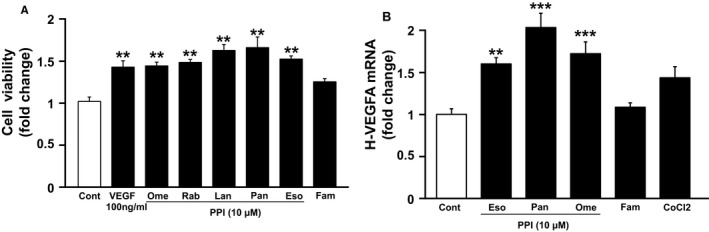
Effect of proton pump inhibitors to cell survival and promotion of angiogenesis. (A) Cell viability in HUVEC(n = 5) and (B) h‐VEGFA mRNA expression in LS174T(n = 7) treated with PPIs or Famotidine or CoCl2 at 100 µM for 24 h. Ome; omeprazole, Eso; esomeprazole, Pant; pantoprazole and Famo; famotidine. Data are expressed as mean fold change from controm±SEM (**p < 0.01, ***p < 0.001).

The influence of PPI on *VEGF* gene expression was evaluated using human vascular endothelial cells (HUVEC). Exposure of PPIs (omeprazole, rabeprazole, lansoprazole, pantoprazole, and esomeprazole; 100 μM) increased cell proliferation significantly in HUVEC, as did the exposure of *VEGF*. However, there were no significant increases with exposure of famotidine. Thus, the effect of PPI on VEGF‐A mRNA expression was then evaluated using LS174T cells. VEGF‐A mRNA expression was significantly increased by PPI exposure in LS174T cells. However, there was no significant increase in exposure of famotidine (Figure 1). Cobalt Chloride (CoCl_2)_ was used as positive control.

## 3 DISCUSSION

In this study, we identified drugs that reduced the occurrence of hypertension in patients treated with bevacizumab and then investigated whether these drugs could reduce the efficacy of bevacizumab. We used FAERS to identify that among patients treated with bevacizumab, there were fewer reports of hypertension in patients taking omeprazole. However, the other PPIs did not show an association with the reduction in the occurrence of hypertension in patients treated with bevacizumab. This may be due to the long period of time since omeprazole was approved and the high number of reports compared with the number of adverse event reports for other PPIs in the FAERS database. For large databases such as FAERS, it is difficult to achieve unsupervised collection results and some data may be inaccurate.^20^, ^21^ Furthermore, there are no data on individual patient characteristics. Therefore, we retrospectively investigated the medical records of patients in our hospital.

In the clinical records study, we examined the association between the use of PPIs and response rates. There was a 25% (though not statistically significant) reduction in the ORR and 21% reduction in the disease control rate in patients using PPIs. Thus, there was a reduction in the response to bevacizumab combination chemotherapy. There were no significant differences between the two groups for factors such as stage, PS, or combination regimen.

Although FAERS analysis showed a reduced rate of bevacizumab‐induced hypertension in patients using PPIs, but there was no significant reduction in the chart reviews. This may be because many patients in our hospital had developed hypertension before bevacizumab was administered and were already satisfactorily treated with antihypertensive drugs. Furthermore, the PPI group might have had lower numbers of hypertensive patients, which may have prevented them from achieving significant results. This study suggests that PPI not only reduces the incidence of hypertension in patients treated with bevacizumab but also reduces the efficacy of this drug. However, this study did not examine the duration of PPI dosing, and further investigation is needed to determine the extent to which PPI‐use influences the effects and adverse events of bevacizumab.

We investigated the molecular mechanisms by which the combination of PPI and bevacizumab was found to be associated with poor antitumor effect. We analyzed the GEO databases and found that PPI induces *VEGF* gene expression. It has also been previously suggested that drugs that inhibit *VEGF* expression reduce VEGF‐induced angiogenesis.^23^ This suggests that PPI might reduce the therapeutic effect of bevacizumab by inducing the expression of *VEGF*.

Therefore, we then investigated the effects of PPIs on tumor growth and angiogenesis using the human cell lines. We found that PPIs promoted cell proliferation in the vascular endothelial cell line HUVEC. The inhibition of angiogenesis was related to the mechanism of tumor growth inhibition by bevacizumab,^24^, ^25^ suggesting that the promotion of angiogenesis by PPIs in tumor tissues might have led to a lower response rate in patients using PPI. Next, using the colon cancer cell line LS174T, we revealed that PPIs promoted the expression of the VEGF mRNA expression in the tumor cells. These results indicated that PPI induced *VEGF* expression in tumor cells might have promoted the angiogenesis and cancer cell growth. This could be the mechanism that led to the reduced therapeutic efficacy of bevacizumab in the medical records study. This effect may also vary according to cancer type and should be further investigated for other types of cancer. However, it has been reported that PPIs have an enhanced antitumor effect in pancreatic cancer.^26^ Therefore, for anti‐tumor agents other than bevacizumab, PPIs may have a synergistic effect by mechanisms other than *VEGF*.

There are possible limitations in this study. FAERS analysis includes reporting bias and unmeasured confounding factors. Furthermore, not all adverse events observed in clinical practice are included in the database. Under reporting or selective reporting might be included. Because the FAERS database contains missing data, incorrect drug names, and duplicate data, we removed or corrected such data before the analysis. The FAERS analysis showed that some variables were also limited. We also did not consider race, duration of treatment, drug dosage, or co‐administration of other medications. Although hypertension may have occurred prior to treatment as an underlying condition, the treatment time and the other medications were not listed in the FAERS database. While these aforementioned factors might affect the results, the use of self‐reporting systems can be useful in detecting potential drug signals. Moreover this was a single‐center study and was limited to a specific type of cancer, thus, a multicenter study with a larger cohort is required. Additionally, the relationship between the PPI and the treatment effect of bevacizumab has not been examined because not enough cases have been collected for evaluation. Therefore, further studies are needed to conclude the possibility of PPIs decreasing the therapeutic effect of bevacizumab. Furthermore, further studies, including multicenter and randomized trials, may be considered.

However, we should never dismiss the use of PPIs. PPIs are very important in many diseases such as gastric ulcers and reflux esophagitis. However, the long‐term use of PPIs may be a risk factor for stomach cancer,^27^, ^28^ and shorter prognosis for lung cancer patients receiving immune checkpoint inhibitors.^29^, ^30^ Thus, although PPIs are currently effective in gastric conditions and are frequently used, they may not be used properly.^31^, ^32^ It may be necessary to avoid long‐term administration of PPI which should be administered to only those who truly require it.

In conclusion, the use of PPIs might reduce the antitumor activity of bevacizumab in patients receiving bevacizumab.

## 4 MATERIALS AND METHODS

### 4.1 Analysis of the FAERS database

The FAERS data sets for the third quarter of 2010 to the second quarter of 2015 were obtained from the FDA website (https://www.fda.gov/Drugs/GuidanceComplianceRegulatoryInformation/Surveillance/AdverseDrugEffects/ucm082193.htm). Following the FDA’s recommended method, we used the most recent case numbers to exclude duplicate reports (out of a total of 4331802 reports) and analyzed the remaining 3724555 reports. The definition of hypertension was based on the 10 terms from the Standardized Medical Dictionary for Regulatory Activities (MedDRA) Queries [Hypertension] (SMQ code: 20000147), published in MedDRA/J ver 19.0, by the International Council for Harmonisation.

FAERS reports were classified into four groups: (a) received bevacizumab and reported hypertension, (b) received bevacizumab but did not report hypertension, (c) did not receive bevacizumab but reported hypertension, and (d) did not receive bevacizumab and did not report hypertension. The ROR was used to signal detection and was calculated using the following equation.^16^, ^33^

$$\begin{array}{c} \text{Calculation for ROR}:\text{ROR}=\left(a/b\right)/\left(c/d\right),\\ 95\%\text{CI}=\exp\left(\log\left(\text{OR}\right)\pm 1.96\;\text{square}\;1/a+1/b+1/c+1/d\right) \end{array}$$

Letters a, b, c, and d indicate the number of individuals in each group. If the lower limit of the 95% CI was >1, it was assumed that there was a relationship between the drug use of interest and hypertension report. Conversely, if the upper limit of the 95% CI <1, we assumed that there was an association between the drug use of interest and reduced hypertension report.

### 4.2 Analysis of the medical chart review

In this retrospective study, we investigated patients’ medical records. This data was collected from the medical records of patients who visited our hospital. Patients aged ≥18 years who were diagnosed with colorectal cancer at The Tokushima University Hospital between January 2010 and December 2015 and were treated with bevacizumab combination XELOX, or mFOLFOX6 for the first time, and patients with evaluated lesions using Response Evaluation Criteria in Solid Tumors (RECIST) were included. Patients who were treated with <1 course of bevacizumab, failed to follow‐up, and had missing values were excluded. This study was approved by the Ethics Committee of Tokushima University Hospital (Approval Number: 2962‐1).

The following data were extracted from patient charts: age at diagnosis, sex, cancer type, stage at diagnosis (using the 6th edition AJCC system), Eastern Cooperative Oncology Group PS, bevacizumab dose, treatment duration, blood pressure, and PPI‐use. Patients were considered to have received concomitant PPIs if they had been prescribed PPIs continuously during the bevacizumab treatment period. Blood pressure was measured by the nurse prior to administration of bevacizumab for each cycle. The event of hypertension in patients receiving bevacizumab was defined according to the Common Terminology Criteria for Adverse Events v 4.0 (CTCAE v 4.0): an increase in blood pressure of <20 mmHg, an increase in blood pressure to ≥150/100 mmHg, or hypertension requiring initiation or increase in therapeutic medication. Those who were taking antihypertensive medication before bevacizumab treatment were considered “taking antihypertensive medication” and those who started antihypertensive medication after bevacizumab treatment were considered “not taking antihypertensive medication.” The stage of cancer was extracted from the medical records, and relapse cases were considered as stage IV.

Treatment response was assessed according to RECIST version 1.0 as follows: CR, PR, SD, and PD. The primary endpoint was assessed using the ORR, which is the number of patients achieving CR or PR divided by the number of patients evaluated. Disease control was defined as the number of patients achieving CR, PR, and SD divided by the number of patients assessed.

### 4.3 Analysis of the GEO and KEGG PATHWAY databases

Microarray gene expression data were obtained from the National Center for Biotechnology Information (NCBI) GEO database (http://www.ncbi.nlm.nih.gov/geo/) (accession number GSE59927).We analyzed expression data using samples analyzed in the livers of rats receiving oral omeprazole (30 mg/kg, once daily/5 days) or vehicle (carboxymethylcellulose), focusing on genes involved in the VEGF signaling pathway extracted from the KEGG PATHWAY database (entry number: MAP04370 http://www.genome.jp/dbget‐bin/www_bget?pathway:map04370). The expression levels of each gene were shown in relation to controls by inverting the log2‐transformed microarray data to the original scale.

We chose a rat model and not a human model for this analysis because in reality, the patients were treated with a number of drugs in combination. Thus, it was ethically difficult to administer PPIs alone to humans, and to obtain tissue samples for comparison before and after PPI administration. Therefore, to exclude the effects of concomitant drugs, in vivo experiments were needed to investigate the effects of these drugs.

### 4.4 Real‐time PCR and cell proliferation assay

The LS174T (human colon cancer cells) and HUVEC were cultured in α‐MEM supplemented with 5% fetal bovine serum and 10 mg/mL of 1% (v/v) penicillin streptomycin (Sigma Aldrich, MO, USA). All cells were incubated at 37°C in a humidified atmosphere containing 5% (v/v) CO_2_.

HUVEC were cultured in 96‐well plates and then exposed to PPI: omeprazole, esomeprazole, pantoprazole, rabeprazole, lansoprazole (100 μM), famotidine (100 μM), CoCl_2_ (100 μM), and DMSO (0.1% v/v) for 24 hours, followed by incubation at 37°C in a humidified atmosphere containing 5% (v/v) CO_2_. 3‐(4,5‐dimethyl‐2‐thiazolyl)‐2,5‐diphenyl‐2H tetrazolium bromide (MTT) was then added, and the cell proliferation rate was measured.

LS174T cells were used to investigate gene expression. Cells were seeded in 6‐well plates and incubated for 24 hours, then exposed to PPI: esomeprazole, pantoprazole, omeprazole, (10 μM), famotidine (100 μM), CoCl_2_ (100 μM), and DMSO (0.1% v/v) for 24 hours. Total RNA was extracted from cells using ISOGEN (NIPPON GENE) according to the manufacturer's protocol. Total RNA was reverse transcribed to cDNA using Prime Script^TM^ RT reagent Kit with gDNA Eraser (TaKaRa). The cDNA product was used as a template for Polymerase Chain Reaction (PCR) amplification in a total volume of 10 µl, and the PCR condition were as follows: 95°C for 2 minutes, followed by 40 cycles at 95°C for 5 seconds, 60°C for 30 seconds, and 95°C for 5 seconds. Reactions were conducted on a CFX96 Real‐time PCR Detection System (Bio‐Rad). All data were analyzed with the CFX Manager Software (Bio‐Rad). The data were analyzed using the delta cycle threshold method and calculated based on the Cq values; the expression of each gene was normalized to 36B4. Gene‐specific primer pairs were used: h‐VEGFA: (F) 5'‐AGGAGGAGGGCAGAATCATC‐3’, (R) 5'‐ATGTCCACCAGGGTCTCGAT‐3’; 36B4: (F) 5'‐GCTCCAAGCAGATGCAGCA‐3’, (R) 5'‐CCGGATGTGAGGCAGCAG‐3'.

### 4.5 Statistical analysis

For FAERS analysis, Microsoft Access 2013 was used for the FAERS database construction and R ver 3.2.1 (R Foundation for Statistical Computing, Vienna, Austria) was used for statistical analysis using the Fisher's exact test. For the retrospective chart review, the Mann‐Whitney U‐test was used as the distribution was not normal. Differences between categorical values were estimated using the chi‐square or Fisher's exact test for comparisons with cells <5. Data obtained from the pathway analysis of RT‐PCR experiments were performed using the Dunnett's test. All recorded P values were two‐sided, and P values <0.05 were considered significant.

The continuous variable case records are shown as mean ± standard deviation. The demographic characteristics were evaluated with descriptive statistics. Student's *t* test with Welch's correction was used when the data distribution was normal, and the Mann‐Whitney *U*‐test was used when the distribution was not normal.

## CONFLICT OF INTEREST

The authors have no conflicts of interest directly relevant to the content of this article.

## AUTHOR CONTRIBUTIONS

YZ is the principal investigator and guarantor of the paper, had full access to all the data in the study, and takes responsibility for the integrity of the data and the accuracy of the data analysis. KY designed the research and drafted the manuscript. MM, TN, YI, MG, MC, KF, TS, SI, TS, NO, HH, YH, YI, HY, and KI contributed to the acquisition, analysis, or interpretation of data and critically reviewed and revised the article for important intellectual content. All authors approved the final manuscript and decided to submit the article for publication.
